# Tribological Behavior of Copper–Graphite Composites Reinforced with Cu-Coated or Uncoated SiO_2_ Particles

**DOI:** 10.3390/ma11122414

**Published:** 2018-11-29

**Authors:** Haohao Zou, Xu Ran, Weiwei Zhu, Yong Wang, Siqi Zhan, Zhikang Hao

**Affiliations:** Key Laboratory of Advanced Structural Materials, Ministry of Education, Changchun University of Technology, Changchun 130012, China; haoozou@163.com (H.Z.); wynn1209@163.com (Y.W.); zsqsqq@163.com (S.Z.); hao123zansqsqq@163.com (Z.H.)

**Keywords:** tribological behavior, copper matrix composites, coating, silica, brake

## Abstract

Copper–graphite composites reinforced with SiO_2_ particles were fabricated by powder metallurgy technique. Electroless copper plating was introduced to improve the interfacial bonding between SiO_2_ particles and copper matrix. The microstructure, density, and hardness of the composites were characterized. The tribological properties, such as friction coefficient and wear rate of the composites, were studied using a pin-on-ring tribometer. The results show that the hard SiO_2_ can restrict the severe plastic deformation and adhesion contact in the process of wear. At the same time, parts of SiO_2_ particles can be broken into fine particles during wear process, which is helpful for decreasing adhesion wear and abrasive wear. Therefore, the addition of SiO_2_ leads to increasing friction stability and friction coefficient, and decreasing wear rate. In addition, the electroless copper plating improves the interfacial bonding between SiO_2_ and copper matrix, which prevents separation of SiO_2_ from copper matrix and further increase tribological properties of the composites.

## 1. Introduction

Copper matrix composites are wildly used as brake materials of high speed vehicles, such as aircraft and high speed train, owing to its good machinability and excellent thermal conductivity [1,2,3,4]. Graphite as solid lubricant often is added into the copper matrix friction materials to stabilize the friction coefficient and reduce wear rate during sliding by forming a graphite-rich transfer layer on the counterpart surface [5,6,7]. However, the addition of soft graphite leads to the decrease of mechanical properties and friction coefficient of the composites [8,9,10,11,12,13,14].

The brake materials require not only good lubricating property but also excellent mechanical properties and high friction coefficient. Ceramic particles—such as Al_2_O_3_, SiC, SiO_2_, and TiC—with high hardness, wear resistance, melting point, and thermal stability can be used as reinforced phase to improve the mechanical and tribological properties of copper matrix composites [15,16,17,18]. Among those ceramic particles, SiO_2_ exhibits many advantages, such as relatively low prices, near zero thermal expansion, and relatively less abrasive effect on the composites. Previous studies have reported that SiO_2_ particle could improve the mechanical properties and wear properties of metal matrix composites [19,20,21,22,23,24]. However, the weak interface bonding between ceramic particle and metal matrix probably results in pulling out of ceramic particle from the matrix during the brake process and deteriorating the tribological properties. In particular, the detached SiO_2_ particle may cause severe abrasive wear. Rohatgi et al. [23] reported that the higher amount of SiO_2_ particle deteriorates the tribological properties of Al matrix composites due to the poor interface bonding between SiO_2_ particle and Al matrix. 

Metallization of ceramic particle is a common method to improve the interfacial bonding between ceramic particle and the metal matrix [25,26]. For example, Lee et al. [27] fabricated Cu/SiC_p_ composites with Cu-coated or uncoated SiC particles. They found that SiC particles coated with a copper layer can improve the interfacial bonding strength between SiC particles and copper matrix and enhance the mechanical properties of the composites. However, to the best of our knowledge, no tribological properties of copper matrix composites reinforced with coated ceramic particles have been reported and the tribological mechanism is not clear. 

In this study, SiO_2_ particles were coated with a copper layer by electroless method. Copper–graphite composites reinforced with Cu-coated or uncoated SiO_2_ particles were fabricated by the powder metallurgy technique. The tribological properties, the friction and wear mechanism of the composites were studied using a pin-on-ring tribometer at different sliding speed and applied load.

## 2. Experimental

**Figure 1:**
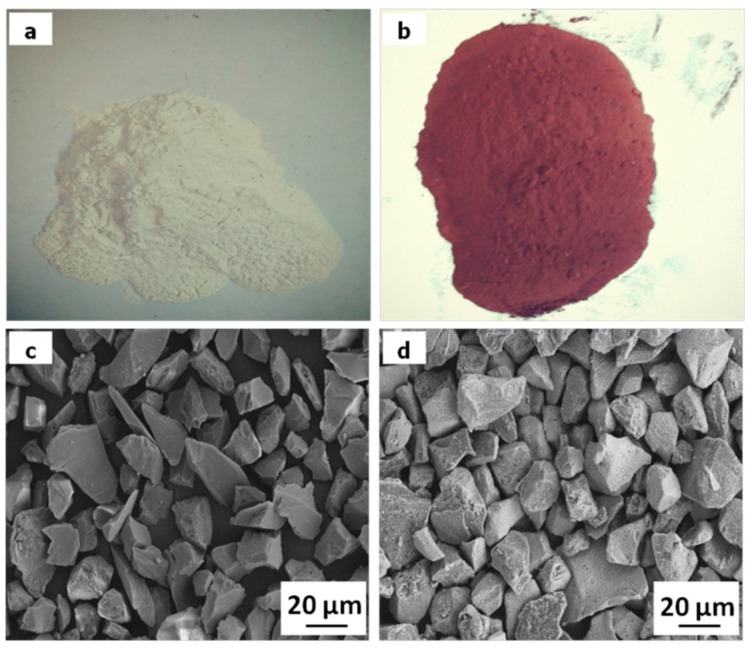
Macro and micro morphologies of SiO_2_: (**a**,**c**) uncoated; (**b**,**d**) Cu-coated.

Electrolytic Cu powder (purity >99.9 wt %, 45 μm in diameter, density of 8.92 g/cm^3^), flaky graphite (purity >99.5 wt %, 200–300 μm in diameter), and SiO_2_ particles (purity >99.5 wt %, size of 10–20 μm, density of 2.2 g/cm^3^) were used for fabricating the composites. The SiO_2_ particles were coated with copper up to about 60 mass % using electroless method. The composition of the electroless bath is given in Table 1. The macro and micro morphologies of SiO_2_ particles before and after electroless plating are shown in Figure 1. It is observed that after electroless process, a uniform and continuous Cu layer is coated on the surface of SiO_2_ particles. At the same time, the color of SiO_2_ particles is similar to that of copper.

Copper–graphite–silica composites were fabricated by powder metallurgy technique. A high-frequency mixer (GQM-2-5, Wuxi Jialong Equipment Technology Co., Ltd, Wuxi, China) with a rotation speed about 400 rpm was used to mix copper powder, flaky graphite and silica particles. The composition of the composites is shown in Table 2. Then these mixtures were cold compacted by plate vulcanizer (XLB, Qingdao Yadong Machinery Group Co., Ltd, Qingdao, China) at a load of 500 MPa to achieve a green specimen. Sintering was performed at 900 °C for 1h under a uniaxial pressure of 2 MPa by using a vacuum-hot-press-sintering furnace (HVRY-I, Shenyang Yite Electrical Apparatus Co., Ltd, Shenyang, China).

The actual density of the composites was tested based on Archimedes’ law. The relative density was calculated by the ratio of actual density to theoretical density. The hardness was measured using Brinell hardness (HBW) by applying a load of 500 N for 15 s. The hardness tests were carried out at least five different locations for each sample and the average value was reported. The microstructures of composites were characterized by scanning electron microscope (SEM, JSM-5500LV, JEOL Ltd., Tokyo, Japan).

**Figure 2:**
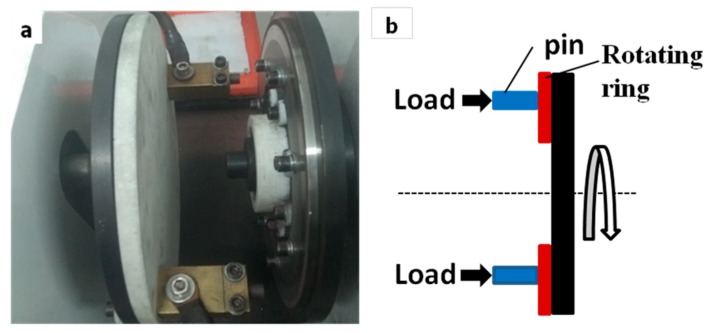
Pin-on-ring tribometer illustration: (**a**) test machine; (**b**) schematic of pin-disc tribometer.

The friction and wear tests were carried out using a pin-on-ring tribometer (FHC-05, Jinan Hengxu Testing Machine Technology Co., Ltd, Jinan, China), as shown in Figure 2. A ring with an outer diameter of 240 mm, inner diameter 160 mm, and thickness 8 mm was made by 30CrMoV with a hardness of 60 ± 2 HRC. The diameter and height of the tested pins is 10 mm and 13 mm, respectively. Before each test, the specimens were finished by emery papers to obtain an average surface roughness of 0.4 μm (Ra), and then cleaned with acetone to remove any surface contaminants. The wear and friction behavior were investigated at load of 0.4, 0.6, 0.8, and 1 MPa, sliding speed of 3, 6, 9, and 12 m/s, and the sliding distance of 12,000 m. The distance between the two pins is 200 mm. The tests were performed under unlubricated conditions at room temperature.

The wear lost was measured according to the change in weight of the pins before and after the test. The wear rate was measured as a function of the wear lost divided by the sliding distance. The coefficient of friction as a function of the sliding distance was calculated and recorded in the computer by sensors. The friction coefficient values were calculated by using the formula μ = F/N, where μ is the friction coefficient, F is the frictional force in Newtons, and N is the normal force in Newtons.

The morphologies of worn surfaces and wear debris were observed by scanning electron microscope (SEM). The chemical composition of worn surfaces was examined by energy dispersive spectroscopy (EDS, EDAX-Falcon, EDAX Inc., Mahwah, NJ, USA).

## 3. Results

### 3.1. Microstructure, Density, and Hardness of Composites

**Figure 3:**
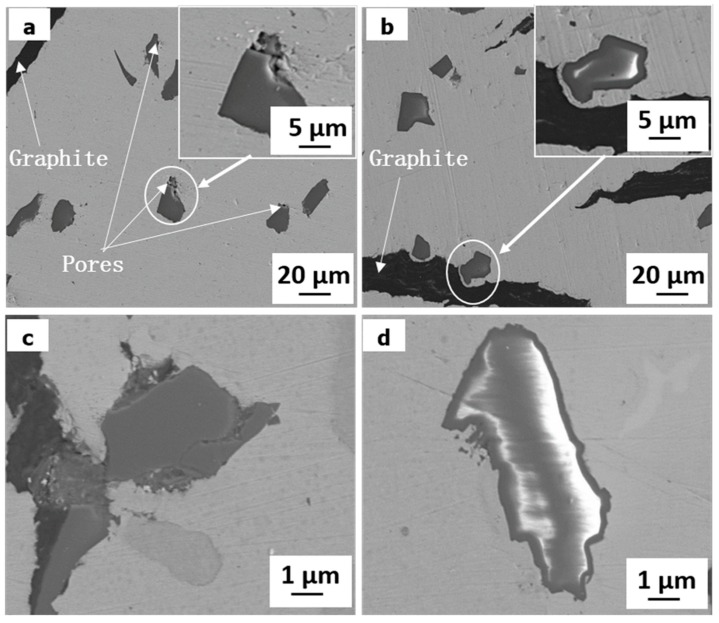
SEM morphologies of the composites: (**a**,**c**) uncoated SiO_2_; (**b**,**d**) Cu-coated SiO_2_.

Figure 3 shows the SEM morphologies of specimens reinforced with Cu-coated and uncoated SiO_2_, respectively. Some pores can be observed in the interface between SiO_2_ particles and copper matrix for specimen reinforced with uncoated SiO_2_, as shown in Figure 3a,c. However, it can be seen from Figure 3b that there is a good bonding between SiO_2_ and copper in specimen reinforced with Cu-coated SiO_2_. The encapsulation of SiO_2_ with Cu results in good contacts between SiO_2_ particles and copper matrix during sintering. Therefore, it is effective to improve the interfacial bonding between SiO_2_ and Cu by electroless copper plating.

The density and hardness of the composites are presented in Table 3. It is observed that the relative density decreases with the increase of SiO_2_ particles for uncoated SiO_2_ particles reinforced composites, due to the formation of pores. However, the coating of SiO_2_ particles with Cu before sintering is helpful to increase the relative density of composites. The hardness of the composites increases with the increase of SiO_2_ content. It means that SiO_2_ particles play a good dispersion strengthened effect. In addition, the hardness of specimens 1S-Cu and 3S-Cu is higher than that of specimens 1S and 3S, respectively. This should be attributed to the well bonding of SiO_2_ particles with copper matrix and high relative density in specimens 1S-Cu and 3S-Cu.

### 3.2. Friction and Wear Characteristics

**Figure 4:**
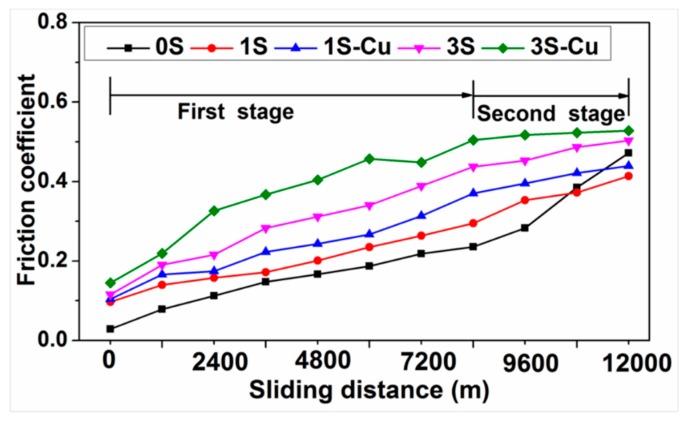
Variation of friction coefficient with sliding distance at applied load of 0.4 MPa and sliding speed of 9 m/s.

Figure 4 shows the typical variation of friction coefficient with sliding distance at applied load of 0.4 MPa and sliding speed of 9 m/s. The wear process can be divided into two stages [28,29]. At the first stage of wear test, the friction coefficient of all specimens increases with the increasing of sliding distance. At this stage, the removing of surface oxide layer leads to the metal-to-metal contact, which causes the increasing of friction coefficient [30]. At the same time, the formation of wear debris also enhances the friction resistance of worn surface [31]. At the second wear stage, a rapid increase of friction coefficient can be observed in specimen 0S. This is because the temperature of worn surface rises with the increase of sliding distance, which results in softening and plastic deformation on the worn surface of specimen 0S. The plastic deformation leads to asperity junctions between the counterparts [32]. However, specimens 1S, 3S, 1S-Cu, and 3S-Cu exhibit a slow increase of friction coefficient value. Further, the friction coefficient of specimens 1S-Cu and 3S-Cu is more stable than that of specimens 1S and 3S, respectively. This should be attributed to the hard particles restricting the plastic deformation of composites by imbedding in the matrix and bearing most of the wearing force [33].

**Figure 5:**
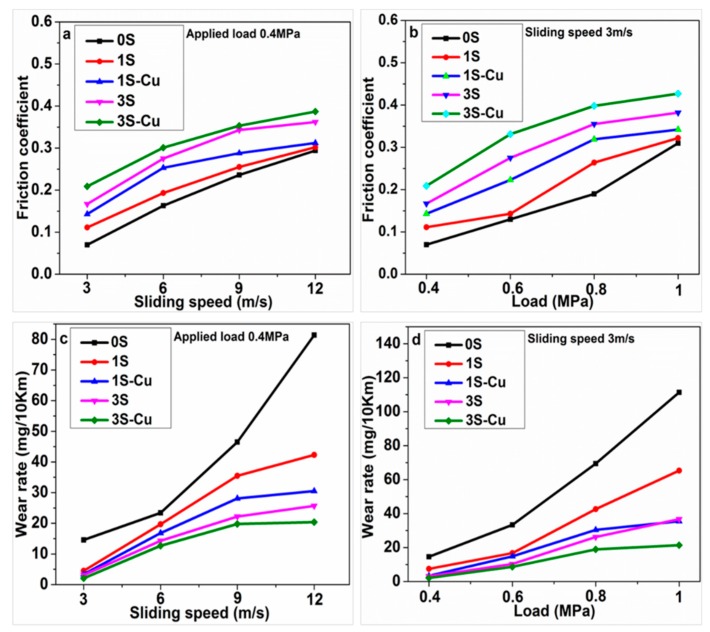
Variation of average friction coefficient (**a**,**b**) and wear rate (**c**,**d**) with sliding speed (**a**,**c**) and applied load (**b**,**d**).

Figure 5a,b shows the variation of average friction coefficient of the composites with sliding speed and applied load, respectively. This is an agreement with the results obtained by Ma and Lu [32] and Prabhu [22]. With the sliding speed and applied load increase, more friction heat generated on the worn surface, and the temperature of worn surface rises. Figure 5c,d shows the variation of wear rate of composites with sliding speed and applied load, respectively. It can be found that the friction coefficient of composites increased and the wear rate decreased with the increasing of SiO_2_ content at all friction condition. Furthermore, specimen 1S-Cu and specimen 3S-Cu has a higher friction coefficient and lower wear rate than specimen 1S and 3S, respectively. 

### 3.3. Worn Surface and Wear Debris

**Figure 6:**
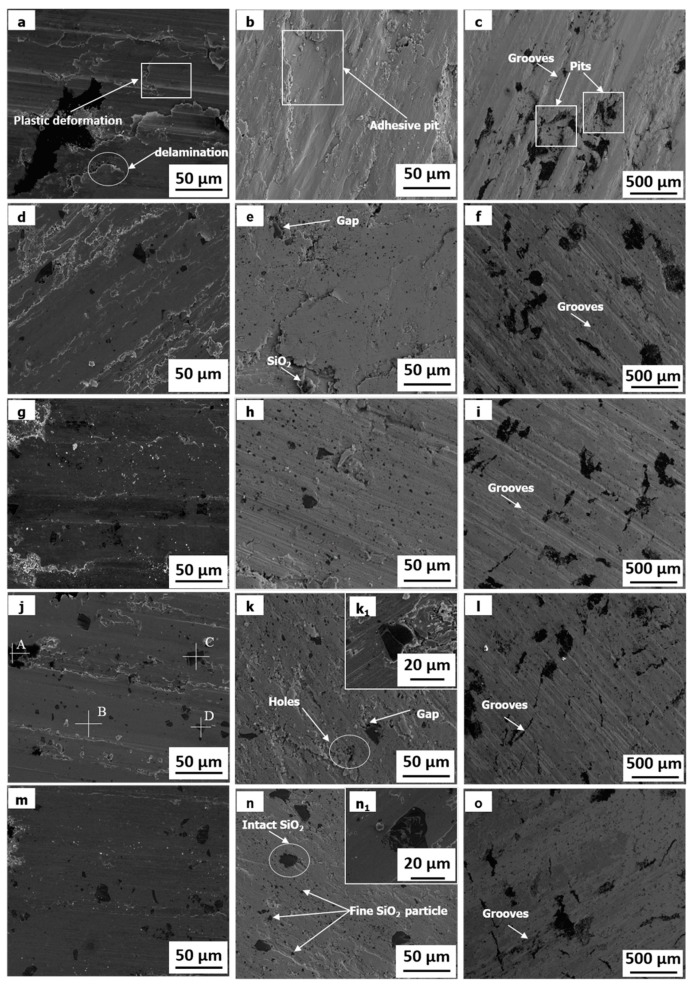
SEM morphologies of the worn surfaces of 0S (**a**–**c**), 1S (**d**–**f**), 1S-Cu (**g**–**i**), 3S (**j**,**k**,**k1**,**l**), and 3S-Cu (**m**,**n**,**n1**,**o**) at sliding speed of 3 m/s and applied load of 0.4 MPa (**a**,**d**,**g**,**j**,**m**) and 0.8 MPa (**b**,**c**,**e**,**f**,**h**,**i**,**k**,**k1**,**l**,**n**,**n1**,**o**).

Figure 6 shows the SEM morphologies of the worn surface of specimens at sliding speed of 3 m/s and applied load of 0.4 MPa and 0.8 MPa. The typical chemical composition of the worn surface was shown in Table 4. A large number of plowing grooves, delamination, adhesive pits, and severe plastic deformation can be observed on the worn surface of specimen 0S, as shown in Figure 6a–c. It indicates the wear mechanism of specimen 0S is abrasive wear, delamination, and adhesion wear.

With the content of SiO_2_ particles increase, the worn surfaces of specimens become smoother. Only plowing grooves, slightly plastic deformation and delamination can be found on the worn surface of specimen 1S, 1S-Cu, 3S, and 3S-Cu, as shown in Figure 6d–o. It indicates that the hard SiO_2_ particles can restrict the plastic deformation and adhesion wear of composites. Therefore, the wear mechanism of specimen1S, 1S-Cu, 3S, and 3S-Cu is mainly abrasive wear and delamination wear.

In addition, for composite with SiO_2_ as reinforcement, much fine SiO_2_ particles can be found on the worn surface. Especially, with the increase of applied load, the amount of fine SiO_2_ particles becomes more and more. This indicates that the big SiO_2_ particles can be broken into fine particles during wear process. Meanwhile, the broken SiO_2_ can be embedded into the soft copper matrix (see Figure 6d,e,g,h,j,k,m,n). However, the exfoliation of SiO_2_ particles can be found for the composite with uncoated SiO_2_ as reinforcement. For example, the separated SiO_2_ particles can be observed on the worn surface of specimen 1S (see Figure 6e), the holes are noted on the worn surface of specimen 3S (see Figure 6k). Finally, it can be seen from Figure 6(k1,n1) that there is a big gap between SiO_2_ and copper matrix in specimen 3S. However, no obvious pores can be found between SiO_2_ and copper matrix in specimen 3S-Cu.

**Figure 7:**
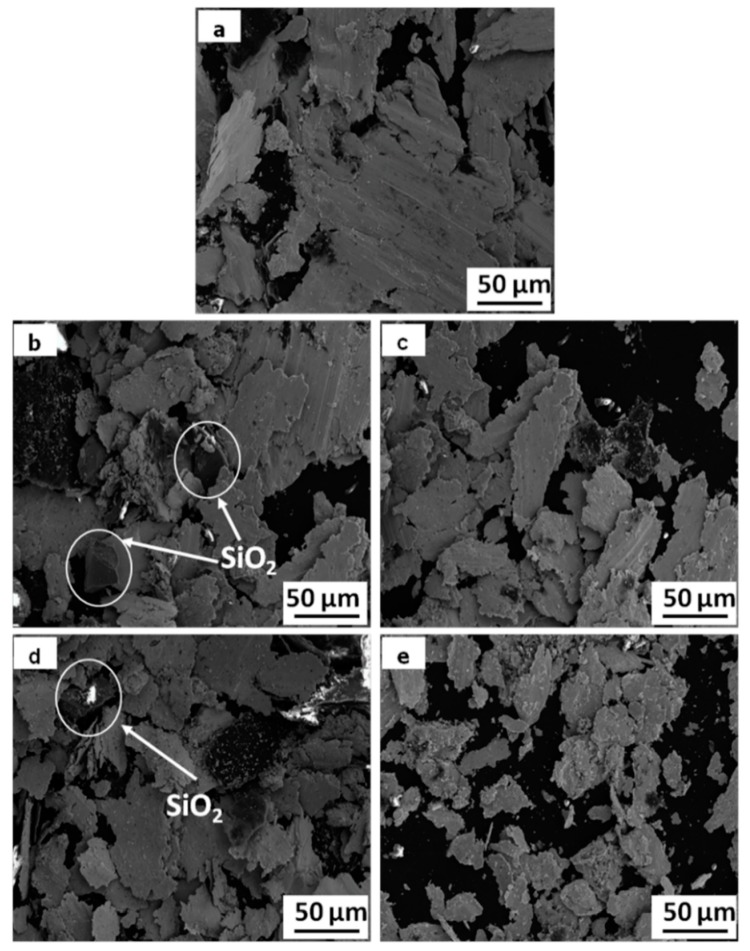
SEM morphologies of wear debris of the composited at 9 m/s and 0.4 MPa: (**a**) 0S; (**b**) 1S; (**c**) 1S-Cu; (**d**) 3S; (**e**) 3S-Cu.

Figure 7 shows the SEM morphologies of wear debris of the composited at applied load of 0.8 MPa and sliding speed of 9 m/s. With the content of SiO_2_ particles increases, the size of wear debris becomes more and more small. In addition, there are some SiO_2_ particles in the wear debris of specimens 1S and 3S. This is further evidence that the SiO_2_ particles without copper plating easily pull out from matrix and removed from worn surface during wear test.

## 4. Discussions

### 4.1. Effect of SiO_2_

It is well known that the SiO_2_ particles possess higher hardness than the Cu matrix. During the wear process, the harder SiO_2_ particles are exposed to the worn surface, which impedes the relative sliding of counterpart and leads to the increasement of friction coefficient. At the same time, the addition of SiO_2_ also increases the hardness of composite (Table 3). According to the empirical Archards’ model [34,35], the wear resistance is proportional to the hardness of the wearing body. Therefore, the wear rate decreases with the addition of SiO_2_ particles. 

Furthermore, from the point of view of wear mechanism, the hard SiO_2_ particles can restrict the severe plastic deformation and adhesion wear based on the observation of wear surface. However, the addition of hard ceramic particles may cause serious abrasive wear. For example, Alpas and Embury [36] reported that the addition of SiC particles to the 2014 Al alloys caused a marginal increase in the wear rate. However, in this work, it can be found that parts of big SiO_2_ particles can be broken into fine particles during wear process. Therefore, no serious abrasive wear is observed for the SiO_2_ reinforced composite. At the same time, these fine particles increase the contact area of SiO_2_-ring, which further hinders the deformation and adhesion contact of the material during the sliding process [37,38]. Therefore, the addition of SiO_2_ can increase the friction coefficient, decrease the wear rate, and improve the friction stability of composites.

### 4.2. Effect of Cu Coating

According to the Figure 3, it can be concluded that the Cu coating is helpful for improving the interfacial bonding between the SiO_2_ particles and copper matrix. For the specimens 1S and 3S, the detached SiO_2_ particle can be observed at the wear surface (Figure 6e) and in the wear debris (Figure 7b,d). It indicates that the SiO_2_ particles without copper coating can be pulled out from matrix and removed from worn surface during wear test due to the poor interface bonding. This can decrease the function of SiO_2_ particles. On the contrary, for specimens 1S-Cu and 3S-Cu, excellent interfacial bonding between Cu-coated SiO_2_ particles not only improves the density and hardness, but also restrains the extraction of SiO_2_ particles from the copper matrix.

### 4.3. Wear Process

**Figure 8:**
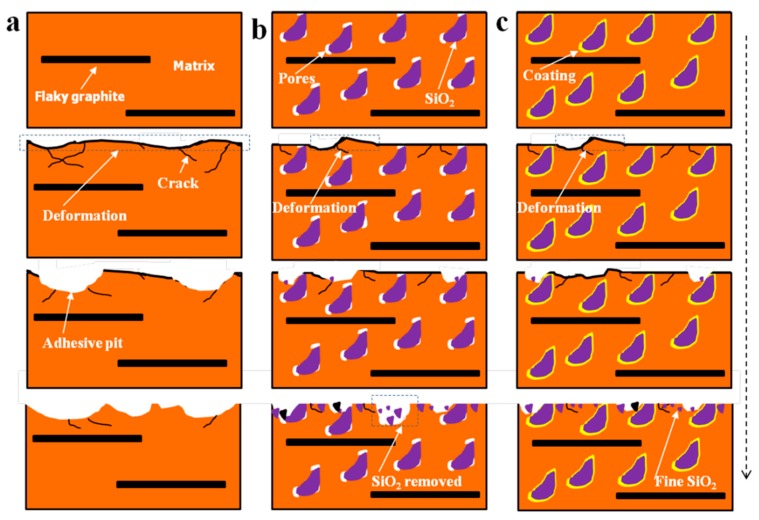
Schematic diagram of the wear process of the composites: (**a**) 0S; (**b**) 3S; (**c**) 3S-Cu.

The schematic diagram of the wear process of composites is illustrated in Figure 8. With the sliding distance increase, the temperature of worn surface rise lead to soften of the composites. Under the combined action of shearing stress and normal force, plastic deformation appeared, and thermal stress cracks initiation for all specimens. 

For specimen 0S, as the thermal stress cracks grow, large block of materials break off from the composites, resulting in big wear pits on the worn surface, as shown in Figure 8a. For specimen 3S, SiO_2_ particles can carry the part of normal load [18]. When the cracks encounter SiO_2_ particles, they may end or spread around from the SiO_2_ particles. Both of that result in a relatively slight plastic deformation of worn surface and low wear rate. Meanwhile, the hard SiO_2_ particles exposed on the worn surface can cut the spalling blocks into fine wear debris (see Figure 7a,b,d), this may decrease the adhesion contact of the sliding counterpart. In addition, a part of SiO_2_ particles fracture and embed into the soft copper matrix. The dispersion distribution of broken SiO_2_ particles further decreases the metal–metal contact and adhesion wear. However, due to the poor interfacial bonding between uncoated SiO_2_ particles and copper matrix, some SiO_2_ particles is easy to be pulled out from copper matrix. For specimen 3S-Cu, the excellent interfacial bonding between Cu-coated SiO_2_ particles and copper matrix, restrains the extraction of SiO_2_ particles from the copper matrix. Therefore, composites reinforced with Cu-coated SiO_2_ particles have a lower wear loss.

## 5. Conclusions

Copper–graphite composites reinforced with Cu-coated or uncoated SiO_2_ particles were fabricated by the powder metallurgy technique. The microstructure, density, hardness, and tribological properties of the composites were characterized. The conclusions are drawn as follows:

(1) The hardness of the composites increases with the increase of SiO_2_ content. However, the poor interfacial bonding between SiO_2_ particles and Cu matrix, result in the relative density decreased with the increase of SiO_2_ particles for uncoated SiO_2_ particles reinforced composites. Electroless copper coating is helpful to increase the relative density of composites by improve the interfacial bonding between SiO_2_ particles and copper matrix.
(2) The addition of SiO_2_ lead to increasing of friction stability, friction coefficient, and decreasing of wear rate. This should be attributed to the hard SiO_2_ can restrict the severe plastic deformation and adhesion contact in the process of wear.
(3) The electroless copper plating improve the interfacial bonding between SiO_2_ and copper matrix, which helps to prevent SiO_2_ pull out from copper matrix and further increase tribological properties of the composites.

## Figures and Tables

**Table 1:** Technology conditions of electroless copper coating.

CuSO_4_·5H_2_O	EDTA·2Na	KNaC_4_H_6_O_6_	HCHO (Solution)	NaOH	Temperature
16 g/L	25 g/L	20 g/L	14 mL/L	14 g/L	40 °C

**Table 2:** Composition of the composites.

Sample	Copper (mass %)	Flaky Graphite (mass %)	Silica (mass %)
0S	97	3	0
1S	96	3	1
3S	94	3	3
1S-Cu	95.4	3	1 + 0.6 (weight of coating)
3S-Cu	92.2	3	3 + 1.8 (weight of coating)

**Table 3:** Density and hardness of the composites.

Sample	Relative Density	Brinell Hardness (HBW)
0S	93.50%	39.8 ± 0.5
1S	92.55%	40.6 ± 0.9
3S	91.76%	41.4 ± 1.1
1S-Cu	93.10%	41.2 ± 0.6
3S-Cu	93.03%	43.7 ± 1.0

**Table 4:** Chemical composition (at %) of the worn surface.

Point	Si	O	Cu	C	Possible Phase
A	-	0.8	10.3	88.9	Graphite
B	-	8.1	90.7	1.2	Cu matrix
C	30.3	63.2	6.2	0.3	SiO_2_
D	29.6	61.5	8.5	0.4	SiO_2_
